# Brain Function Network and Young Adult Smokers: A Graph Theory Analysis Study

**DOI:** 10.3389/fpsyt.2019.00590

**Published:** 2019-08-30

**Authors:** Ying Tan, Jing Chen, Weiwei Liao, Zhaoxin Qian

**Affiliations:** ^1^Health Management Center, Xiangya Hospital, Central South University, Changsha, China; ^2^Center of Stomatology, Xiangya Hospital, Central South University, Changsha, China; ^3^Department of Emergency, Xiangya Hospital, Central South University, Changsha, China

**Keywords:** young smokers, brain functional networks, graph theory analysis, resting-state functional magnetic resonance imaging, topological organization

## Abstract

Cigarette smoking is associated with abnormalities in the widespread inter-regional functional connectivity of the brain. However, few studies focused on the abnormalities in the topological organization of brain functional networks in young smokers. In the current study, resting-state functional magnetic resonance images were acquired from 30 young male smokers and 32 age-, gender-, and education-matched healthy male nonsmokers. A functional network was constructed by calculating the Pearson correlation coefficients among 246 subregions in the human Brainnetome Atlas. The topological parameters were compared between smokers and nonsmokers. The results showed that the functional network of both young smokers and nonsmokers had small-world topology. Compared to nonsmokers, young smokers exhibited a decreased clustering coefficient (C_p_) and local network efficiency (E_local_). C_p_ and E_local_ were negatively correlated with the duration of cigarette use. In addition, increased nodal efficiency (E_nodal_) was mainly located in the prefrontal cortex (PFC), cingulate gyrus, insula, and caudate. Decreased connectivities among the PFC, cingulate gyrus, insula, basal ganglia (of specific node), and thalamus were also observed. In sum, we revealed the abnormal topological organization of brain functional networks in young smokers, which may improve our understanding of the neural mechanism of young smokers from a brain functional network topological organization perspective.

## Introduction

Cigarette smoking is associated with serious health problems, such as brain-, heart-, and lung-related diseases (1). The majority of lifelong smokers and those with higher levels of nicotine dependence are people who started smoking at an early age (2–5). Structural and functional deficits have been found in the brains of young smokers (6–9). The abnormal cortical thickness and gray matter volume/density mainly in the frontal cortex, anterior cingulate cortex, and striatum have been detected in young smokers (6, 10, 11). In addition, the resting-state functional connectivity (RSFC) among the dorsolateral prefrontal cortex (PFC), orbitofrontal cortex (OFC), and striatum were significantly reduced in smokers (6, 12, 13). These regional and circuit-level structural and functional abnormalities in young smokers (13–15) may contribute to whole-brain functional network changes. Thus, the main purpose of the current study is to detect the changes in the topological organization of brain functional networks in young smokers.

Modeling the human brain as a complex network has provided a powerful mathematical framework to characterize the functional architecture of the brain (16). Graph theory analysis (GTA) provides a powerful mathematical framework for characterizing the intrinsic topological organization in the whole-brain functional networks, such as the small-word property, connectivity, global efficiency (E_global_), and E_nodal_ (16–18). The small-world network is characterized by a distinctive combination of high clustering coefficient (C_p_) and short characteristic path length (L_p_), which can quantify efficient information segregation and integration and may facilitate the spread of information in networks (19). The brain functional network’s efficiency can be quantitatively calculated by GTA and characterize the capacity for parallel information transfer at the brain node and global levels (20). GTA has been applied to the study of brain-related diseases in both brain structural and functional networks in addiction research (18, 21–24). Less efficient network architecture and disruptions in the topological organization of brain networks had been observed in smokers (25). Similarly, heroin-dependent patients showed decrease normalized C_p_ (γ) and small-worldness in the brain functional networks compared to health controls (22). GTA has provided novel insights into the neurobiological mechanisms for young smokers. Clear evidence showed that the number of parcellations will impact the local characteristics and metrics of the brain network architecture. As the number of nodes increased, an increased stability of network metrics could be observed (26). Previous studies constructed the structural and functional networks with 90 regions of interest from the Automated Anatomical Labeling template as network nodes (21–24, 27). To increase the accuracy of the brain function network analysis based on GTA, in the current study, we employed 246 regions in the human Brainnetome Atlas—a detailed fine-grained, cross-validated atlas that contains information on both anatomical connectivity and FC patterns—to parcellate gray matter into distinct regions (28).

Therefore, in this study, we aimed to compare the differences in topological organization of brain functional networks (246 regions) between nonsmokers and young smokers. Based on previous addiction findings on brain functional network topography, we hypothesized that (1) differences in the global and node topological parameters should be detected between nonsmokers and young smokers; (2) compared to nonsmokers, young smokers showed significant FC among the nodes in the PFC, striatum, and insula; and (3) abnormal topological properties of the brain functional network may be related to smoking behaviors in young smokers. We hoped that our findings could improve our understanding of the neurobiological mechanisms by revealing the brain functional network organization in young smokers.

## Materials and Methods

All procedures of the present study were approved by the Medical Ethical Committee of the Xiangya Hospital of Central South University. All participants and their parents gave written informed consent after the experimental procedure was fully explained.

### Participants

Thirty young male smokers (mean age, 21.2 ± 1.15 years) and 32 matched healthy male nonsmokers (mean age, 20.8 ± 1.08 years) were recruited from local universities (**Table 1**). In the current study, all young smokers met the diagnostic criteria of nicotine dependence in the Diagnostic and Statistical Manual of Mental Disorders, Fifth Edition, and have not abstained from smoking for longer than 3 months since they had started smoking. The nicotine dependence level of young smokers was assessed by the Fagerström Test for Nicotine Dependence (FTND). The cumulative amount of nicotine intake was indexed by pack-year. Nonsmokers were recruited from people who smoked no more than five cigarettes in their lifetime. None of the subjects have physical illness (brain tumor, obstructive lung disease, hepatitis, or epilepsy), neurological disease (e.g. stroke), or claustrophobia according to clinical evaluations and medical records. None of the subjects reported daily consumption of alcohol, drug abuse, or dependence (other than nicotine dependence for young smokers) or current medication use that may affect cognitive functioning. The carbon dioxide level in expired air, as measured by the Smokerlyzer system (Bedfont Scientific Ltd., Rochester, UK), was verified as 3 ppm in nonsmokers and ≤8 ppm in the abstinence state, showing a distinct reduction for young smokers compared to those not currently smoking.

**Table 1:** Demographic characteristics of nonsmokers and young smokers in this study.

Items	Smokers (*n* = 30)	Controls (*n* = 32)	*t*	*p*
Age (years)	21.2 ± 1.15	20.8 ± 1.08	–1.254	0.215
Levels of education (years)	14.07 ± 0.58	14.09 ± 0.53	0.191	0.849
CPD	15.6 ± 5.10	—	—	—
Age at smoking onset	15.7 ± 2.50	—	—	—
Years of smoking	4.5 ± 2.47	—	—	—
Pack-years	3.7 ± 2.95	—	—	—
FTND	4.8 ± 1.66	—	—	—

### Image Acquisition

This experiment was carried out on a 3 T magnetic resonance imaging (MRI) system (Signa; General Electric) with an eight-channel phase-array head coil at Xiangya Hospital of Central South University. Participants were asked to refrain from smoking for 60 min immediately preceding the scan. All the subject’s heads were positioned carefully with comfortable support, and earplugs were used to reduce scanner noise. The resting-state functional images were obtained with the following parameters: 30 contiguous slices with a slice thickness of 5 mm; repetition time = 2,000 ms; echo time = 30 ms; flip angle = 90°; field of view = 240 × 240 mm^2^; data matrix = 64 × 64; and total volumes = 180. During the entire scan, subjects were instructed to keep their eyes closed, relax their minds, but not fall asleep and remain as motionless as possible.

### Data Preprocessing

Statistical Parametric Mapping software (SPM8; http://www.fil.ion.ucl.ac.uk/spm) was used to perform data preprocessing. Data preprocessing included the following steps. First, the initial 10 volumes were discarded for magnetization equilibration for each dataset. Second, slice-timing correction and head-motion correction were performed in the remaining volumes to exclude the subjects with a maximum displacement greater than 1.5 mm or head rotation greater than 1.5°. Third, corrected images were normalized to the Montreal Neurological Institute space and resampled to a 3 mm isotropic voxel. The nuisance signal regression was carried out by including the six motion parameters, their first-order temporal derivatives, white matter, and ventricular cerebrospinal fluid signal (14-parameter regression). Finally, a temporal band-pass filter (0.01–0.08 Hz) was applied to reduce the effects of high-frequency physiological noises.

### Brain Functional Network Construction

#### Network Node Definition

To define the nodes of the network, we chose 246 regions (210 cortical and 36 subcortical subregions) for whole-brain parcellation, providing a fine-grained, cross-validated atlas and containing information on both anatomical and functional connections (28). Each brain region represented a node of the functional network. This set of 246 putative functional regions was shown to more accurately represent the information present in the network relative to voxel-wise and atlas-based parcellation approaches (29). The detailed information can be found http://atlas.brainnetome.org/.

#### Network Edge Definition

To determine the edges of the network, the regional mean time series of all possible pairs of 246 brain regions were evaluated for Pearson correlation coefficients and *z*-transformed correlation coefficients to generate 246 × 246 whole-brain FC matrices for each subject. Then, the absolute *z* correlation matrices were thresholded to obtain binarized matrices [i.e., adjacency matrices A = (aij)] according to a threshold range of 0.10 < cost < 0.40 with intervals of 0.01 (25) to avoid correlation-level differences between groups (30). Cost was defined as the ratio of the existing number of edges to the maximum possible number of edges in a network. The entry aij was 1 when the absolute *z*-value between regions i and j exceeded the threshold and 0 otherwise.

#### Network Analysis

The topological properties of the brain functional networks were explored for each threshold, including small-world parameters [γ, normalized characteristic path length (λ), and scalar small-worldness (σ)], network efficiency [global level: global network efficiency (E_global_) and local network efficiency (E_local_); regional level: E_nodal_ for each node i as a nodal metric], and global parameters (area under the curve for each network measure, which provides a summarized scalar for topological properties).

#### Small-World Parameters

C_p_ and L_p_ are two important elements of small-world network parameters that were first proposed by Watts and Strogatz (31).

$$C_{p}=\frac{1}{N}\frac{E_{i}}{D_{i}(D_{i}-1)/2},$$

where E_i_ is the number of existing connections within the network and D_i_ is the degree of node i. The C_p_ of a network quantifies the extent of local interconnectivity or cliquishness of the network (31).

$$L_{p}=\frac{1}{N(N-1)}\sum_{i\neq j\in G}L_{i,j},$$

where L_i,j_ is the L_p_ between nodes i and j. The characteristic path length of the network (L_p_) measures the potential for integration between brain regions (31).

The C_p_ and L_p_ of the brain networks were compared to those of random networks to examine the small-world properties. In this study, we defined the normalized L_p_ (λ), $\lambda =L_{p}^{real}/L_{p}^{rand}$, and the γ, $\gamma =C_{p}^{real}/C_{p}^{rand}$. One hundred matched random networks, which had the same number of nodes, edges, and degree distribution as the real networks (32), were generated. $L_{p}^{rand}$ and $C_{p}^{rand}$ are the mean C_p_ values and the mean L_p_ of 100 matched random networks. When a network meets the criteria of γ > 1 and λ ≈ 1 (25) or δ = γ / λ > 1 (33), the network has small-world properties.

#### Network Efficiency

E_global_ measures the efficiency of parallel information transfer in the network (34):

$$E_{global}=\frac{1}{N(N-1)}\sum_{i=1}^{N}\sum_{\underset{j\neq i}{j=1}}^{N}\frac{1}{L_{ij}}$$

E_local_ quantifies the fault tolerance in the network and reveals the communication efficiency among the immediate neighbors of the node i when it is removed (34):

$$E_{local}=\frac{1}{N}\sum_{i\in G}E_{global}(G_{i}),$$

where G_i_ denotes the subgraph composed of the nearest neighbors of node i.

#### Regional Nodal Characteristics

The E_global_ of a node i (E_nodal_) measures the information propagation ability of a node with the rest of the nodes in the network.

$$E_{nodal}=\frac{1}{N-1}\sum_{j\neq i\in G}\frac{1}{L_{i,j}},$$

### Statistical Analysis

#### Comparisons of Demographic Variables

The differences in demographic characteristics between 30 young smokers and 43 nonsmokers were tested with the two-sample *t* test using SPSS 20.0 software.

#### Comparisons of Global Parameters and Regional Efficiency

We detected the significant differences in network topological properties (C_p_, L_p_, E_global_, and E_local_) and E_nodal_ between nonsmokers and young smokers using nonparametric permutation tests. Specifically, the group differences were obtained using a two-sample *t* test with *p* = 0.05. The process for the nonparametric permutation tests was as follows: For each permutation, two sets of data were pooled to create a single set. Then, we selected the actual data of one group from the merged data randomly as the new group, with the rest constituting the other group. Then, the *t* value was calculated and acted as the statistical magnitude. A total of 5000 permutations were performed to obtain an empirical estimate of the null distribution of the differences. We sorted the 5000 recorded differences and computed the proportion of the between-group differences exceeding the differences in real cortical networks. The threshold for statistical significance was *p* < 0.005. The network-based statistical approach was used to localize the abnormal FC about the specific pairs of brain regions in the WM network. Regression analyses were performed to examine the association of the network parameters with duration cigarette of use, controlling for age and years of education as covariate variables. The results were visualized using the BrainNetViewer package (http://www.nitrc.org/projects/bnv).

## Results

### Demographic Information

All of the participants were right-handed males. There was no difference between nonsmokers and young smokers in the distributions of age (*p* = 0.215) or level of education (*p* = 0.849; **Table 1**). According to self-reports, the number of cigarettes per day (CPD) in30 young smokers was 15.6 ± 5.1, the mean FTND score was 4.8 ± 1.66, the mean pack-years were 3.7 ± 2.95, and the mean duration of smoking was 4.5 ± 2.47 years.

### Efficient Small-World Brain Functional Networks

**Figure 1:**
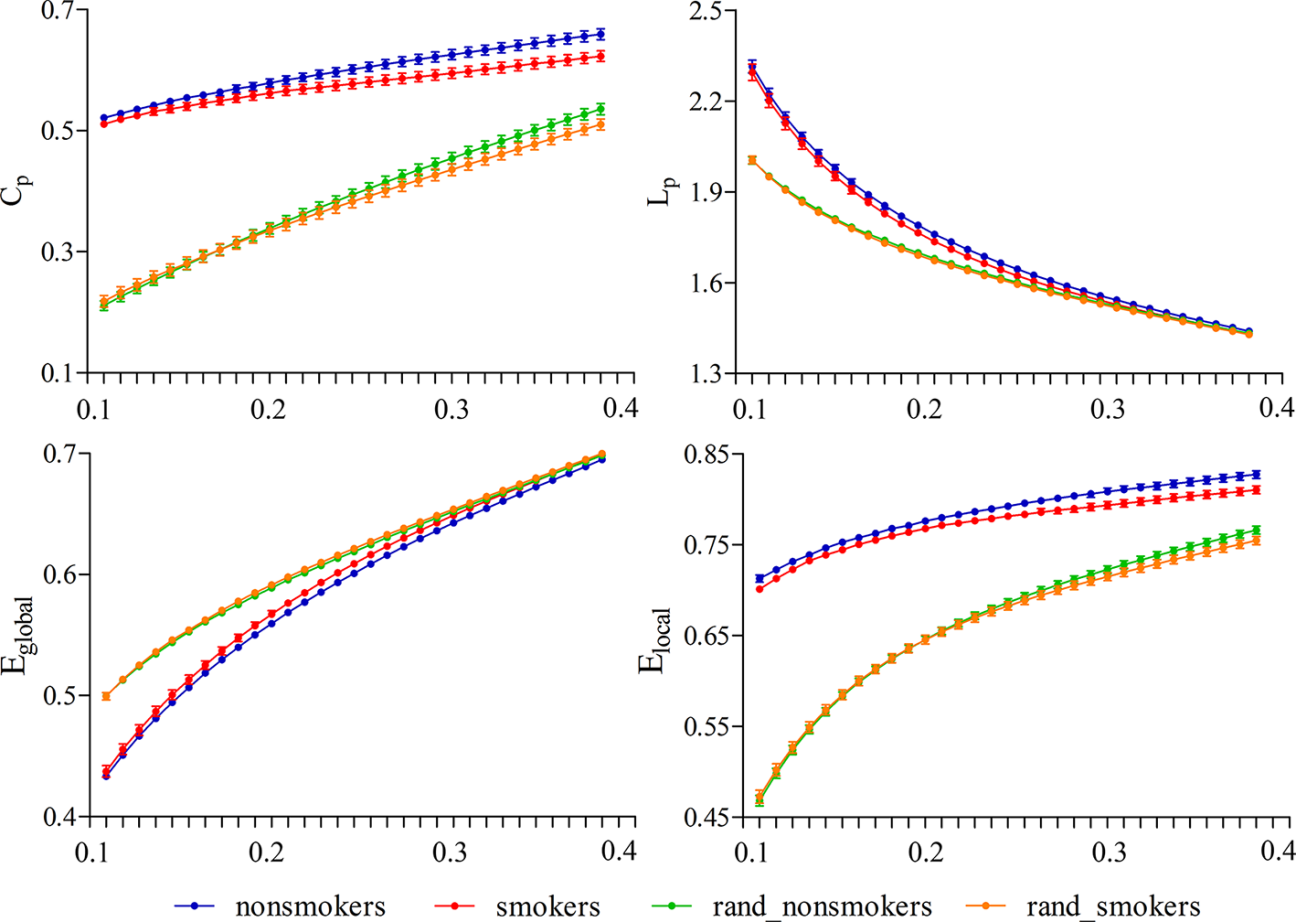
Small-world properties in the entire threshold range of 0.10 < cost < 0.40. All subjects exhibited a higher C_p_ and E_local_ yet have approximately the same L_p_ and E_global_ compared to 100 random networks.

**Figure 2:**
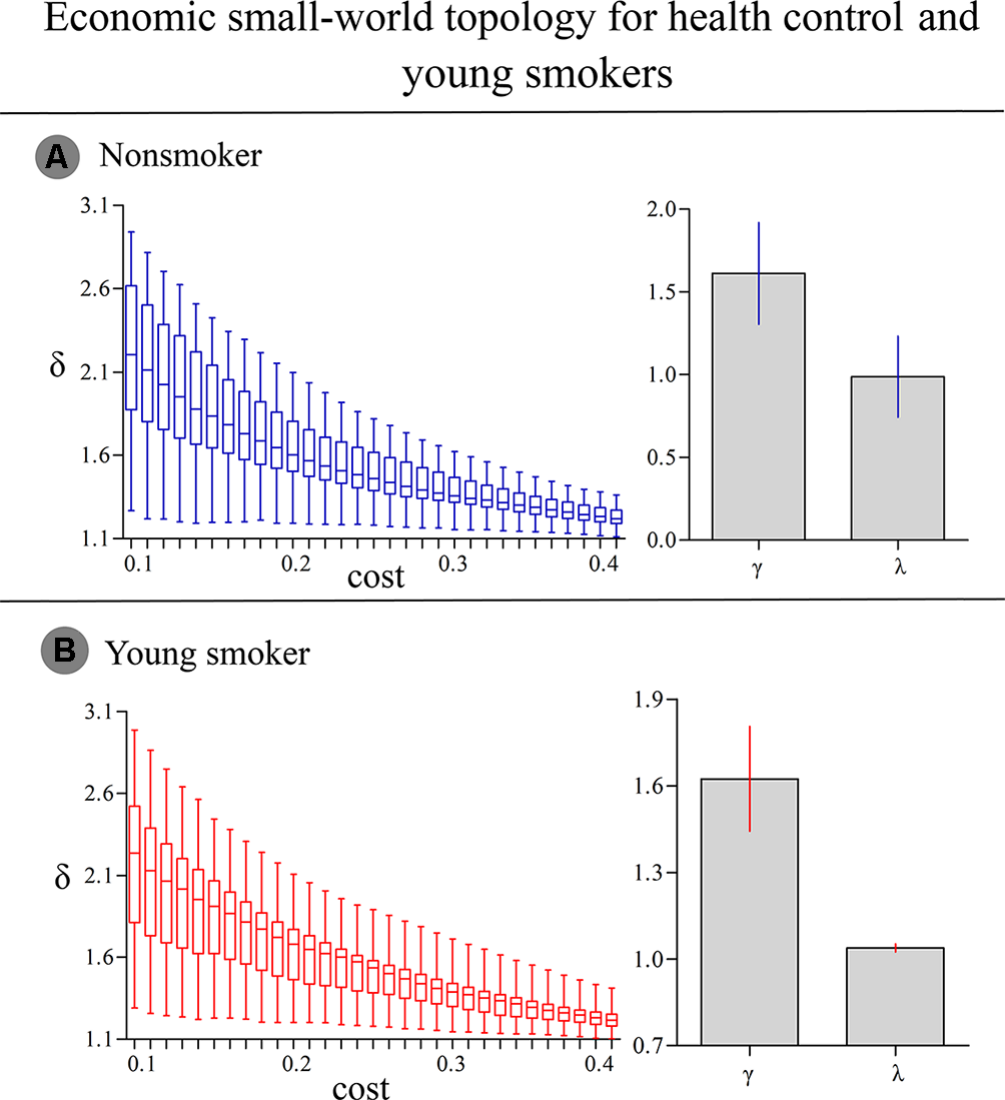
**(A)** In nonsmokers, the σ of each network was larger than 1.1 (δ > 1.1) in the entire threshold range of 0.10 < cost < 0.40. Compared to 100 random networks, all groups had higher γ values (i.e., γ > 1) and nearly identical normalized L_p_ (i.e., λ ≈ 1). **(B)** In smokers, similar δ, γ and λ values were detected.

For nonsmokers and young smokers, their functional networks showed small-world properties in the entire threshold range of 0.10 < cost < 0.40 (**Figures 1** and **2**). Across the wide threshold range (0.10 < cost < 0.40), all the subjects exhibited a higher C_p_ and E_local_ yet had similar L_p_ and E_global_ compared to 100 random networks (**Figure 1**) (31). In addition, compared to 100 random networks, all the groups had higher γ values (i.e., γ > 1) and nearly identical normalized L_p_ (i.e., λ ≈ 1), and the σ of each network was larger than 1.1 (δ > 1.1) for all subjects in the entire threshold range of 0.10 < cost < 0.40 (**Figure 2**).

### Global Topology of Brain Function Networks

**Figure 3:**
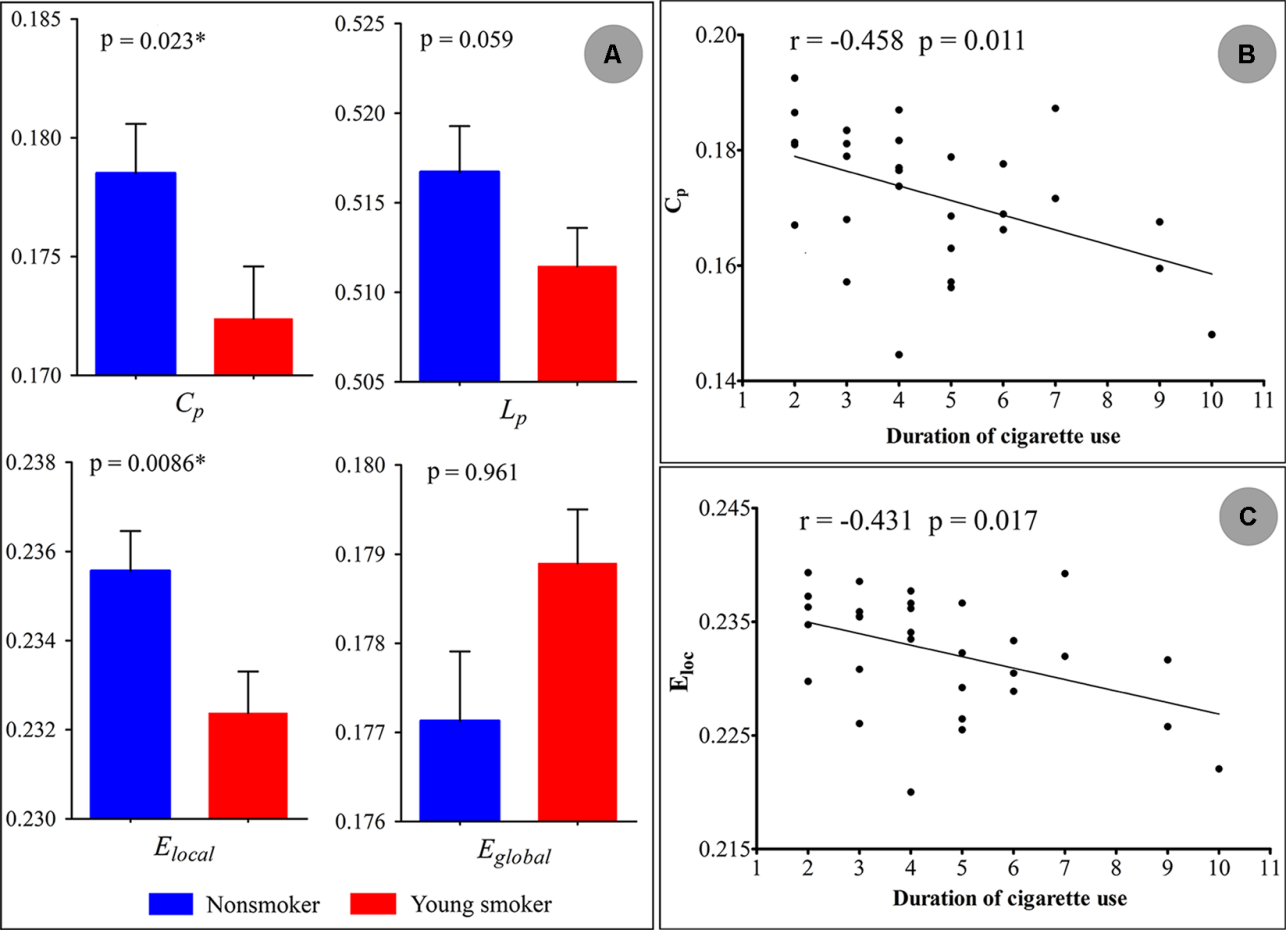
**(A)** Statistical analysis revealed significant differences in the global parameters between the nonsmokers and young smokers. **(B and C)** The clustering coefficient (*C* *_p_*: *r* = −0.458; *p* = 0.011) and local network efficiency (*E* *_local_*: *r* = −0.431; *p* = 0.017) were negatively correlated with duration of cigarette use.

Statistical analysis revealed significant differences in the global parameters between nonsmokers and young smokers (**Figure 3A**). Compared to nonsmokers, young smokers showed a significantly decreased C_p_ (*p* = 0.023) and E_local_ (*p* = 0.0086) (**Table 2**). C_p_ (*r* = –0.458; *p* = 0.011) and E_local_ (*r* = –0.431; *p* = 0.017) were negatively correlated with the duration of cigarette use (**Figures 3B, C**).

**Table 2:** Comparisons of global network measures between nonsmokers and young smokers.

	C_p_	L_p_	E_local_	E_global_
Control	0.1785 (0.011)	0.517 (0.014)	0.235 (0.005)	0.177 (0.004)
Satiety	0.1724 (0.012)	0.511 (0.011)	0.232 (0.005)	0.178 (0.003)
*p*	0.0232*	0.0596	0.0086*	0.9612
*p < 0.05 compared within groups.				

### Nodal Characteristics of Brain Functional Networks

**Figure 4:**
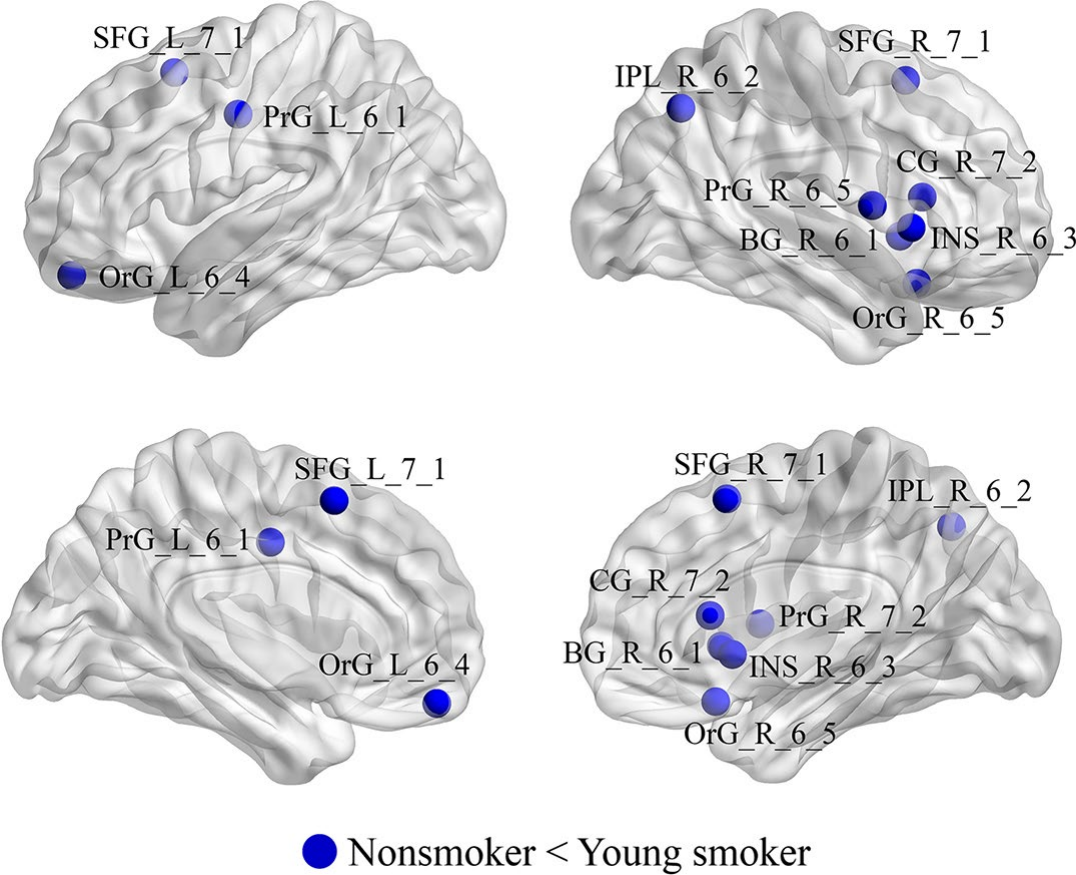
Regions with significant differences in E_nodal_ between young smokers and nonsmokers. The statistical criterion for between-group differences was set at *p* < 0.05 after 10,000 permutation tests.

Compared to nonsmokers, young smokers exhibited significantly increased nodal efficiencies (E_nodal_) in 10 regions (*p* < 0.05 after 5000 permutation tests; **Figure 4**): superior frontal gyrus: SFG_L_7_1 and SFG_R_7_1 (B/A, medial area 8), orbital gyrus: OrG_L_6_4 (B/A, medial area 11) and OrG_R_6_5 (B/A, area 11), precentral gyrus: PrG_L_6_1 (B/A, area 4) and PrG_R_6_5 (B/A, area 4), inferior parietal lobule: IPL_R_6_2 (B/A, rostrodorsal area 39), insular gyrus: INS_R_6_3 (dorsal agranular insula), cingulate gyrus: CG_R_7_2 (B/A, rostroventral area 24), and basal ganglia: BG_R_6_1 (ventral caudate).

### Abnormal Brain FC in Young Smokers

**Figure 5:**
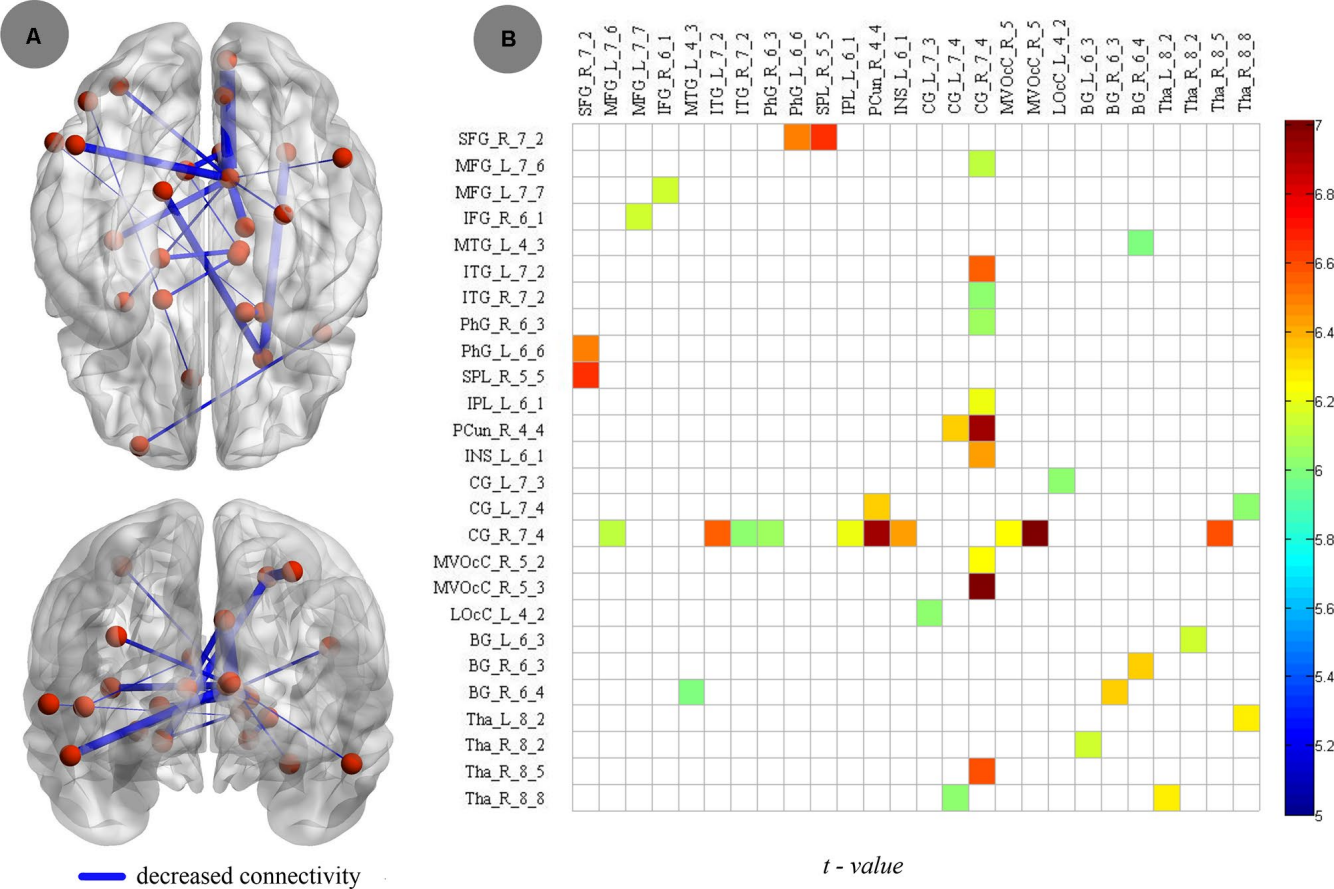
Compared to nonsmokers, young smokers showed decreased connectivity, including 26 brain regions and 20 edges, with two-sample *t* test (*p* < 0.005, Bonferroni correction).

Compared to nonsmokers, young smokers showed increased connectivity, including 26 brain regions and 20 edges, such as the frontal lobe (superior frontal gyrus: SFG_R_7_2, middle frontal gyrus: MFG_L_7_6 and MFG_L_7_7, and inferior frontal gyrus: IFG_R_6_1), temporal lobe (middle temporal gyrus: MTG_L_4_3, inferior temporal gyrus: ITG_L_7_2 and ITG_R_7_2, and parahippocampal gyrus: PhG_R_6_3 and PhG_L_6_6), parietal lobe (inferior parietal lobule: IPL_L_6_1 and precuneus: PCun_R_4_4), insular lobe (insula: INS_L_6_1), limbic lobe (cingulate gyrus: CG_L_7_3, CG_L_7_4 and CG_R_7_4), occipital lobe (medioventral occipital cortex: MVOcC_R_5_2 and MVOcC_R_5_3 and lateral occipital cortex: LOcC_L_4_2), and subcortical nuclei (basal ganglia [BG]: BG_L_6_3, BG_R_6_3 and BG_R_6_4, thalamus: Tha_L_8_2, Tha_R_8_2, Tha_R_8_5, and Tha_R_8_8; **Figure 5A**). Moreover, the *t* values of statistical analyses in the connections between two groups are shown in **Figure 5B**.

## Discussion

In this study, we investigated the topological organization of brain functional networks in nonsmokers and young smokers. The results are as follows: (1) At the global level, the topological organization of brain functional networks in the two groups consistently showed the small-world property, whereas the global topological organization differed significantly between nonsmokers and young smokers — young smokers showed a significantly decreased C_p_ and E_local_. C_p_ and E_local_ were negatively correlated with the duration of cigarette use. (2) At the regional level, changes in nodal E_global_ were mainly found in the frontal gyrus, cingulate gyrus, insula, and BG. (3) At the connectivity level, increased connectivity in young smokers was primarily detected in the frontal gyrus, cingulate gyrus, BG, and thalamus. These findings may improve the understanding of neurophysiologic mechanisms in whole-brain functional networks in young smokers.

### Global Parameters

The brain function networks in both nonsmokers and young smokers showed the small-world property. Small-world organization is one of the most influential findings about human brain functional networks and is a promising characteristic to describe large-scale brain networks. A small-world network has high local C_p_ and L_p_ (35) as well as highly effective local information processing and rapid global information transfer, which supports both specialized processing in local clusters and integrated processing over the entire network (25) and maintains an optimal balance between local specialization and global integration (36). Previous human brain functional network studies have identified the small-world property using various imaging techniques, such as magnetoencephalography, electroencephalography, and functional MRI (19, 22, 24, 37). Consistent with previous studies, in the current study, we observed that both nonsmokers and young smokers showed small-world architecture in the brain functional networks (**Figures 1** and **2**).

Although a small-world topology was detected in both nonsmokers and young smokers’ brain functional networks, there were significant group differences in network global properties. Compared to nonsmokers, young smokers showed significantly decreased global parameters in C_p_ and E_local_ (**Figure 3A**). Moreover, C_p_ and E_local_ were negatively correlated with the duration of cigarette use (**Figure 3B, C**). C_p_ and E_local_ describe the concentrated clustering of local connections and measure local information transmission and processing capacity (38). In the current study, C_p_ and E_local_ were significantly decreased in young smokers, which revealed weaker local information processing capacity and more difficult information transfer with cliquishness in young smokers. Brain structural connectivity (SC) and FC are strongly related in network topologic organization. SC is the basis of FC and can constrain and shape FC patterns across both local and global scales (39). Previous structural network study revealed that E_local_ in young smokers was increased in the whole brain (18). The increased E_local_ in SC and the decreased E_local_ in FC are complementary to ensure the information transmission efficiency in young smokers.

A longer duration of cigarette use caused lower C_p_ and decreased E_local_ of brain networks. These findings suggest that the correlation is a consequence of the length of cigarette smoking during adolescence. However, Lin et al. found contradictory effects of smoking on the brain function in global topological parameters, showing that chronic smokers had higher C_p_ and E_local_ compared to matched nonsmokers (25). The contradictory effects on the brain functional networks may be related subjects’ individual differences, smoking onset age, duration of cigarette use, age, and exposure time to nicotine in young smokers and chronic or heavy smokers. Further research is needed to confirm the validity of the results.

### Nodal Parameters

We found that increased E_nodal_ in young smokers was primarily in the PFC [including superior frontal gyrus, orbital cortex (OFC), and precentral gyrus], cingulate cortex (B/A 24), dorsal insula, and ventral caudate compared to nonsmokers. Previous studies found that the PFC, cingulate cortex, and insula exhibited smoking-related abnormalities in gray matter morphology, such as reduced cortical thickness and gray matter volume and density (10, 40–45). The PFC is related to motivation and cognitive control in addiction (41, 46–48). Additionally, the OFC plays an important role in generating outcome expectancies that guide decision-making in reinforcement devaluation tasks, and the superior frontal gyrus is closely related to cognitive control (49–52). Previous studies showed that cingulate cortex activation is related to cigarette craving in smokers. The insula is connected with the OFC and cingulate cortex (53–55). Moreover, previous studies mentioned that the insula was involved in the craving and showed significant smoking cue-elicited activation in smokers (44, 53, 56). In addition, mounting evidence suggests that the caudate plays a critical role in the craving of smokers, being significantly correlated with craving ratings (57, 58). In the current study, the increased efficiency in PFC, cingulate cortex, insular, and caudate in young smokers may increase the craving to smoke, thus making it difficult for young smokers to halt the development of nicotine dependence and uncontrollable compulsive drug-seeking behavior.

### Connectivity

Neurobiological models of addiction posit that impaired communication between brain regions may contribute significantly to the behavioral deficits of smokers (18). In the current study, we observed the significantly abnormal connectivity in cortical (PFC, cingulate cortex, and insula) and subcortical (BG and thalamus) regions. The primary decreased connectivity was between the cingulate cortex and the PFC, insula, and thalamus. Previous studies found that cingulate cortex activity is associated with cognitive control (59, 60) and that changes in the activity of the cingulate cortex are correlated with the cognitive deficits in smokers (61). The insula is closely related to smoking behavior, such as cognitive control and craving (53). The activation of the insula was correlated with smokers’ ratings of urge to smoke when they were exposed to smoking-related cues or deprived of smoking (62–64). In addition, abnormal SC between the cingulate cortex and insula was also detected and found to be related to impaired cognitive control in adolescents with Internet gaming disorder (65). Our previous studies demonstrated that the PFC plays a critical role in cognitive control deficits in young smokers (13, 66, 67). The decreased FC in cingulate, insula, and PFC found in the current study may demonstrate that the interaction between the two regions plays a critical role in the cognitive impairments of young smokers. We also found decreased FC in the subcortical region between the BG and thalamus. The BG is a part of the nigrostriatal dopamine circuit, which plays an important role in craving and reward processing in addiction (13, 67–69) In addition, in smoking studies, BG was found to mainly regulate the rewarding effect of smoking and motivation to smoke (57, 70). Previous research found that the thalamus is also related to the reward processing and cognitive control functions (71–74). Therefore, the reduced connectivity between the BG and thalamus in young smokers may provide more scientific evidence for the cause of increased craving in young smokers. The main areas of decreased FC were the PFC, insula, cingulate cortex, BG, and thalamus, which are particularly implicated in the cognitive control, reward-seeking, motivation, and compulsive drug intake that have been put forward to explain the phenomenon of addiction.

The novelty of the current study was the choice of 246 regions to build the network. We replicated previous findings by revealing decreased C_p_ and E_local_ in smokers. In addition, we extended a previous study by demonstrating the RSFC among the PFC, caudate, thalamus, and insular in smokers in a higher spatial resolution. Due to the lack of cognitive tasks, the conclusions and hypotheses should be tested in the future. In the current study, we enrolled only male participants, which suggested that the results may only generalize to males. The cross-sectional experiment design cannot separate the results observed in the current study were the consequences or causes of smoking.

## Data Availability

The datasets generated for this study are available on request to the corresponding author.

## Ethics Statement

All of the procedures of the present study were approved by the Medical Ethical Committee of the Xiangya Hospital of Central South University. All of the participants and their parents gave the written informed consent after the experimental procedure was fully explained.

## Author Contributions

YT, WL, and ZQ conceived and designed the experiments. YT, WL, and JC conducted the experiments and collected data. YT analyzed the data. YT and WL wrote the paper.

## Funding

This work was supported by research grants from the Scientific Research Project of Health and Family Planning Commission of Hunan Province, China (B2019179).

## Conflict of Interest Statement

The authors declare that the research was conducted in the absence of any commercial or financial relationships that could be construed as a potential conflict of interest.
